# What is the effect of the Informed Health Choices secondary school intervention on the ability of students in Rwanda to think critically about health choices after one-year follow-up? A cluster-randomized trial

**DOI:** 10.1186/s13063-025-08779-w

**Published:** 2025-05-15

**Authors:** Michael Mugisha, Laetitia Nyirazinyoye, Dieudonne Kayiranga, Clarisse Marie Claudine Simbi, Faith Chesire, Ronald Senyonga, Matt Oxman, Allen Nsangi, Christopher James Rose, Jenny Moberg, Astrid Dahlgren, Margaret Kaseje, Simon Lewin, Nelson K. Sewankambo, Sarah Rosenbaum, Andrew D. Oxman

**Affiliations:** 1 School of Public Health, College of Medicine and Health Sciences, University of Rwanda, KG 11 Ave Gasabo, Kigali, Rwanda; 2 Department of Community Medicine and Global Health, Institute of Health and Society, Faculty of Medicine, University of Oslo, Oslo, Norway; 3 School of Nursing and Midwifery, College of Medicine and Health Sciences, University of Rwanda, Kigali, Rwanda; 4 Tropical Institute of Community Health and Development, Kisumu, Kenya; 5 Department of Medicine, College of Health Sciences, Makerere University, Kampala, Uganda; 6 Centre for Epidemic Intervention Research, Norwegian Institute of Public Health, Oslo, Norway; 7 Faculty of Health Sciences, Oslo Metropolitan University, Oslo, Norway; 8 Department of Health Sciences åLesund, Norwegian University of Science and Technology (NTNU), Ålesund, Norway; 9 Health Systems Research Unit, South African Medical Research Council, Cape Town, South Africa

**Keywords:** Adolescents, Critical health literacy, Health literacy, Informed health choices, Rwanda

## Abstract

**Aim:**

The aim of this study was to evaluate the effects of the Informed Health Choices secondary school intervention on the ability of students in Rwanda to think critically and make informed health choices after 1 year.

**Methods:**

This was a two-arm cluster-randomized trial conducted in 84 lower secondary schools from 10 districts representing five provinces of Rwanda. We used stratified randomization to allocate schools 1:1 to the intervention or control arm. One class in each intervention school had ten 40-min lessons taught by a trained teacher in addition to the usual curriculum. Control schools followed the usual curriculum. The primary outcome was a passing score (≥ 9 out of 18 questions answered correctly) for students on the Critical Thinking about Health Test completed 1 year after the intervention. We conducted an intention to treat analysis using generalized linear mixed models, accounting for the cluster design using random intercepts.

**Results:**

After 1 year, 35 of 42 teachers (83.3%) and 1181 of 1556 students (75.9%) in the control arm completed the test. In the intervention arm, 35 of 42 teachers (83.3%) and 1238 of 1572 students (78.8%) completed the test. The proportion of students who had a passing score in the intervention arm was 625/1238 (50.5%) compared to 230/1181 (19.5%) in the control arm (adjusted odds ratio 7.6 [95% CI: 4.6–12.6], *p* < 0.0001). The adjusted difference in the proportion of students with a passing score was 32.2% (95% CI 24.5–39.8%).

**Conclusions:**

The IHC secondary school intervention was effective after 1 year. However, the size of the effect was smaller than immediately after the intervention (adjusted difference 32.2% vs 37.2%) due to decay in the proportion of students in intervention schools with a passing score (50.5% vs 58.2%).

**Trial registration:**

Pan African Clinical Trial Registry (PCTR), trial identifier: PACTR202203880375077. Registered on February 15, 2022.

**Supplementary Information:**

The online version contains supplementary material available at 10.1186/s13063-025-08779-w.

## Introduction

Over the past decade, the Informed Health Choices (IHC) network has worked on empowering the public to make informed health choices [1]. This is in response to overwhelming misinformation that is available to the public through one-to-one communication, mass and social media, and other sources of communication [1–4]. Exposure to misinformation and acting on poorly informed decisions may lead to harm and waste of resources. Systematic reviews of interventions to teach the public to think critically about health information have found only one small randomized trial of a school-based intervention to teach critical thinking about health to adolescents [5, 6]. To respond to this problem, the IHC network aimed to develop the capacity of young people to think critically about health claims, evidence, and choices. We have developed school-based educational interventions to do this [7, 8].

We initially developed and evaluated educational resources for primary school children [9–11]. The lessons in these resources focused on 12 prioritized key concepts. The key concepts are principles for thinking critically about whether to believe claims about the effects of interventions and for deciding what to do [12]. We also developed and evaluated a podcast for parents of primary school children that focused on nine key concepts [13–15].

Informed by this earlier work, we have developed educational resources for secondary schools. Adolescence is a critical age where young people learn quickly [16]. We developed and evaluated the IHC secondary school intervention in East Africa (Kenya, Rwanda, and Uganda). We started by exploring the context for teaching critical thinking about health in lower secondary schools in East Africa [17–19]. This informed the design of digital learning resources that were fit and scalable in the East African context. We worked with educational stakeholders to prioritize key concepts that were relevant to the targeted age group (13–15 years lower secondary school students) [20]. We employed a human-centered design approach to develop low-cost and scalable digital learning resources in close collaboration with students and teachers in East Africa [21].

To ascertain the effect of the intervention, we conducted randomized trials in Kenya, Rwanda, and Uganda [22–24]. The intervention was effective in all three countries [25]. We conducted process evaluations alongside the three trials and found that the intervention was largely implemented as intended [26–28]. Factors that facilitated effective implementation were that participants valued the content, the design of the resources, and teacher training workshops at the start of the school term when the 10 lessons included in the resources were taught. Factors that limited effective implementation were lack of printed materials for students, especially in schools with fewer computers, competing priorities, and the fact that schools focus on teaching what is prescribed in the curriculum and what is expected to be examined in national evaluations.

Systematic reviews have found little evidence of the effectiveness of educational interventions to teach adolescents to think critically about health [5, 6], and no evidence of the extent to which what is learned is retained. This study aimed to assess effects of the IHC secondary school intervention and retention of what was learned 1 year after the intervention was delivered in Rwanda.

## Methods

### Design

We report the findings after 1 year for a two-arm, cluster-randomized trial conducted in Rwanda between 12 and 23 June 2023. We previously reported findings immediately after the intervention [22]. We received ethical approval from the Rwanda National Ethics Committee (RNEC) (Approval No. 1019/RNEC/2020 and subsequent amendments No. 41/RNEC/2022 and No. 236/RNEC/2022). The trial protocol can be found online [26]. Before data collection, we obtained permission to conduct the study in schools from the Rwanda Basic Education Board. The trial was registered in the Pan African Clinical Trial Registry, trial identifier: PACTR202203880375077.

### Setting and participants

The trial was conducted in lower secondary schools. We included private, public, and government-aided schools that followed the national curriculum. We recruited schools that had over 100 students and 10 teachers, and that had computers and internet. We exclude schools that were hard to reach and schools with a special needs or international curriculum.

We randomly selected 84 schools from 10 districts, two from each of the five provinces in Rwanda. We stratified schools by their performance on national examinations (low or high performance as defined by Rwanda National Examination and School Inspection Authority) and the number of schools was proportionate to the number of schools in each district. We selected one science teacher and one second-year class in each school. Details of the settings, participants, and recruitment process can be found in our previous report [22]. We sought consent and assent from teachers, school directors, and students.

### Random allocation and masking

We allocated schools in a 1:1 ratio to the intervention or control. We used block randomization to balance for school performance, with block sizes of six and four, and equal numbers in each arm. Concealed allocation was conducted by a statistician who was not involved in the recruitment of schools or the analysis of data. We did not change the list after random allocation by the statistician. We did not mask the trial participants or investigators.

### Procedures

The intervention included ten lessons covering nine key concepts [8] taught in a single school term, in addition to the usual curriculum. The lessons were taught using the digital educational resources that we developed [21]. The resources included lesson plans and background information for each lesson, a teachers’ guide, a glossary, and other resources. Each lesson could be taught using a projector, if one was available, or a blackboard. All the schools in the Rwandan trial had projectors. The teachers who taught the lessons attended a 3-day teacher training workshop before teaching the lessons. Participants in the control schools followed the usual curriculum. A detailed description of the intervention is provided using the GREET checklist in Supplementary File S1 [29].

Students and teachers in both control and intervention schools completed the Critical Thinking about Health Test (Supplementary File S2) at the end of the school term and 1 year later. We developed this test to measure the ability of students to understand and apply the key concepts taught in the intervention arm. The questions were taken from the Claim Evaluation Tools item bank [30]. Based on cognitive interviews, we made minor modifications to the questions to ensure they were correctly understood and appropriate. We conducted a Rasch analysis to assess the validity and reliability of the test [31]. We used a combination of the Nedelsky and Angoff methods to determine the cut off for passing and mastery scores [32].

Research assistants administered the test. Research assistants who were not available for the 1-year follow-up were replaced by new ones. We trained all research assistants before data collection. They supervised the test in schools to ensure independent answering. After the test, the research assistant scanned the answer sheets.

### Outcomes

The primary outcome was the proportion of students with a passing score (≥ 9 out of 18 questions answered correctly) on the Critical Thinking about Health Test. Secondary outcomes were the proportion of teachers with a passing score, the proportion of students and teachers with a mastery score (≥ 14 out of 18), students’ and teachers’ mean scores (percent correct answers for the 18 multiple-choice questions), the proportion of students that answered both questions correctly for each of the nine concepts, intended behaviors, and self-efficacy.

### Statistical analysis

We computed the sample size based on the primary outcome, using the University of Aberdeen Health Services Research Unit’s Cluster Sample Size Calculator [33]. We made the following assumptions: 39 students per cluster (one class in each school) based on education statistics [34], an intraclass correlation at 0.19 and 30% of students achieving a passing score in the control arm based on a previous trial in primary schools, a minimally important difference of 20% based on at least 50% of students in the intervention arm having a passing score, an alpha of 1%, power of 90%, and a maximum 10% loss to follow-up. Based on these assumptions, we calculated a sample size of 84 schools.

In the analysis, we estimated adjusted odds ratios and differences in means for binomial and continuous outcomes, respectively. We estimated adjusted odds ratios using mixed effects logistic regression. Adjusted differences in means were estimated using mixed effects linear regression. For outcomes measured at the level of student, we accounted for the cluster-randomized design using random intercepts at the level of school (the unit of randomization). Because there was a one-to-one relationship between teachers and schools, it was not necessary to account for clustering at the level of teachers. Except where noted below, all analyses were adjusted for the variable used in the stratified random allocation (low versus high school performance). To aid interpretation, we re-expressed odds ratios as adjusted differences, accounting for uncertainty of the odds in the control arm as well as the odds ratios. Missing test answers were counted as wrong answers. We followed the intention-to-treat principle throughout: all children and teachers who completed the test were included and analyzed in the arms to which they were allocated. We have reported 95% confidence intervals and two-sided *p* values, where appropriate, throughout. All statistical analyses were performed using Stata 16 (StataCorp LLC, College Station, Texas, USA).

We conducted two prespecified sensitivity analyses to explore the risk of bias due to attrition: inverse probability weighting (IPW) and Lee bounds. We calculated Lee bounds for mean score for students [35, 36]. These analyses provide sharp bounds on treatment effect under conditions in which missing outcomes maximally favor or disfavor the intervention. It was not possible to estimate Lee bounds for the teachers because there was no imbalance in the number of teachers lost to follow-up. To account for the cluster-randomized design for the students, we computed confidence intervals using imputed design effects to inflate the variances of the estimators. A design effect for a particular outcome was imputed as the ratio of the variance of the IPW effect estimate (which does account for the cluster design) to the variance of an estimate from the same model without the random intercepts term. It was not possible to adjust these analyses for the stratification variables.

To put the effect of the intervention in the context of the effect sizes reported for other interventions to improve critical thinking or learning in schools, we estimated Hedges’ *g* (a standardized mean difference) for the adjusted difference in students’ mean scores. This was estimated as the ratio of the adjusted difference to within-cluster standard deviation [37].

We estimated adjusted odds ratios comparing students’ ability to correctly answer both multiple-choice questions for each of the nine concepts and present these results as a forest plot. For questions about intended behaviors and self-efficacy, we report numbers and percentages of students for each response option and estimates of adjusted odds ratios comparing dichotomized responses (e.g., very unlikely or unlikely, versus very likely or likely).

We performed two planned subgroup analyses as described in our trial protocol [22]. In the first, we estimated treatment effects for the primary outcome in schools with high and low performance as defined by National Examination and School Inspection Authority (NESA). In the second, we estimated treatment effects for the primary outcome in students whose English reading proficiency was assessed to be advanced, basic, or lacking. Students who correctly answered all four literacy questions in the Critical Thinking about Health Test were categorized as having advanced proficiency. Students who answered both basic questions correctly and one or both of the advanced questions incorrectly were categorized as having basic proficiency. Students who did not correctly answer both basic questions were categorized as lacking basic reading proficiency. For each subgroup analysis, we estimated odds ratios for the interactions between treatment and the variable defining the subgroups. We report these alongside *p* values testing hypotheses of no interaction.

Lastly, we assessed whether the students who were randomized to the intervention liked the lessons, found them easy, and found them helpful. We report numbers and percentages of students for each response option as well as for dichotomized responses (e.g., liked the lessons a little or very much versus disliked the lessons a little or a lot).

## Results

### Characteristics of trial participants after 1 year

**Fig. 1:**
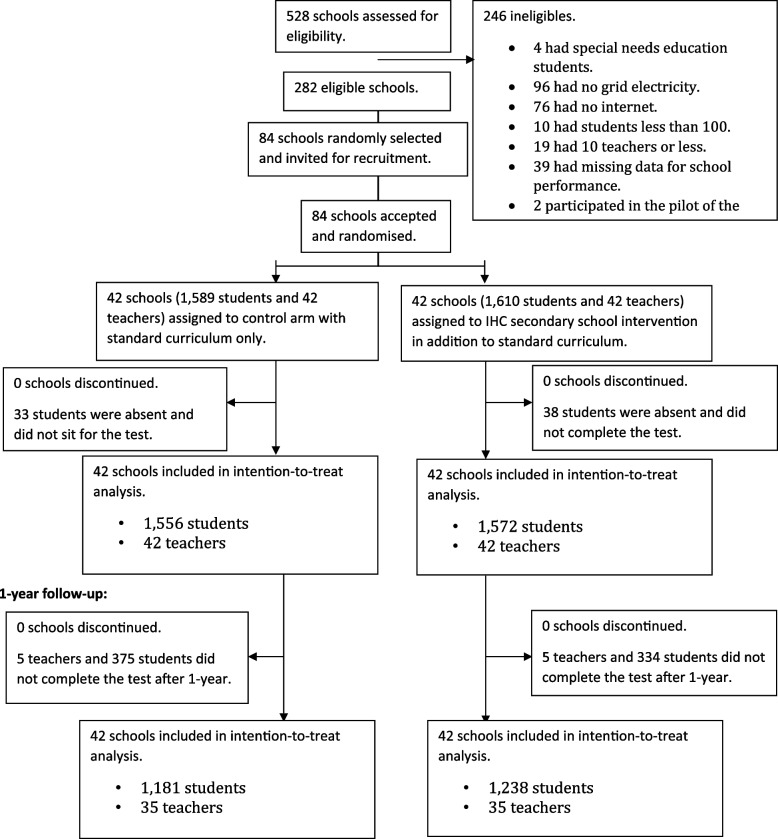
Flow diagram of study participants in 1-year follow-up trial

Between February 25, 2022, and March 29, 2022, we recruited 3128 students in second year of lower secondary and 84 science teachers. We randomly assigned 42 schools (1556 students and 42 teachers) to the control arm and 42 schools (1572 students and 42 teachers) to the intervention arm. All 84 schools participated in the follow-up study. After 1 year, 35 of 42 teachers (83.3%) and 1181 of 1556 students (75.9%) in the control arm completed the test. In the intervention arm, 35 of 42 teachers (83.3%) and 1238 of 1572 students (78.8%) completed the test after 1 year. Figure 1 shows the flow of schools, teachers, and students.

Characteristics of the schools, teachers, and students who participated in the trial are summarized in Table 1. The median number of students per class who completed the test after 1 year was 28 in the control arm and 32 in the intervention arm (compared to 39 and 40 per class at the end of the school term when the intervention was delivered). The percentage of female students who completed the test was 56.3% in the control compared to the 56.2% in the intervention arm (compared to 53.8% and 56.0% at the end of the school term). The mean age of students who completed the test was 15.6 at the time of intervention in both arms (compared to 15.8 and 15.7 at the end of the school term). The number of teachers with bachelor’s degree was 15/42 (35.7%) in control schools compared to 26/42 (61.9%) in intervention arm.

**Table 1:** Characteristics of participants in the trial

		**Control schools**	**Intervention schools**
**School characteristics**			
**Schools**	*N*	42	42
**Province**			
Eastern	*N* (%)	14 (33.3%)	12 (28.6%)
Kigali City	*N* (%)	5 (11.9%)	1 (2.4%)
Northern	*N* (%)	9 (21.4%)	10 (23.8%)
Southern	*N* (%)	9 (21.4%)	7 (16.7%)
Western	*N* (%)	5 (11.9%)	12 (28.6%)
**School type**			
Boarding	*N* (%)	15 (35.7%)	14 (33.3%)
Day schools	*N* (%)	27 (64.3%)	28 (66.7%)
**School ownership**			
Government aided	*N* (%)	19 (45.2%)	26 (61.9%)
Private	*N* (%)	8 (19.0%)	5 (11.9%)
Public	*N* (%)	15 (35.7%)	11 (26.2%)
**School performance**			
Low	*N* (%)	24 (57.1%)	24 (57.1%)
High	*N* (%)	18 (42.9%)	18 (42.9%)
**Completed tests per class**	Median (IQR)	28 (24 to 35)	32 (26 to 38)
**Teacher characteristics**			
**Teachers**	*N*	42	42
**Completed test**	*N* (%)	35 (83.3%)	35 (83.3%)
**Education level**^**a**^			
Advanced diploma	*N* (%)	19 (45.2%)	9 (21.4%)
Bachelor’s degree	*N* (%)	15 (35.7%)	26 (61.9%)
Masters	*N* (%)	1 (2.4%)	0 (0.0%)
**Experience (years)**^**a**^	Mean (SD)	9.2 (5.9)	8.5 (5.7)
**Students’ characteristics**			
**Recruited in the study**	*N*	1556	1572
**Completed test**	*N*	1181	1238
**Gender**^**a**^			
Female	*N* (%)	665 (56.3%)	696 (56.2%)
Male	*N* (%)	516 (43.7%)	542 (43.8%)
**Age**^**a**^	Mean (SD)	15.6 (1.3)	15.6 (1.4)

^a^Data are for participants who took the test

### Main findings of the trial after 1 year

In the intervention arm, 625/1238 (50.5%) of the students had a passing score compared to 230/1181 (19.5%) in the control arm (adjusted odds ratio 7.6 [95% CI 4.6–12.6], *p* < 0.0001) (Table 2). The adjusted difference was 32.2% (95% CI 24.5–39.8%).

**Table 2:** Main results of the primary and secondary outcomes of the trial

	**Control schools**	**Intervention schools**	**Adjusted difference**	**Adjusted odds ratio**	***p*value**	**ICC**
	42 schools 1181 students 35 teachers	42 schools 1238 students 35 teachers				
**Students**						
**Primary outcome**^**a**^						
Students with a passing score (≥ 9/18)^b^	230 (19.5%)	625 (50.5%)	32.2% (24.5 to 39.8%)	7.6 (4.6 to 12.6)	< 0.0001	0.24
**Secondary outcomes**^**a**^						
Students with a mastery score (≥ 14/18)^b^	28 (2.4%)	228 (18.4%)	14.3% (9.5 to 19.1%)	11.7 (5.4 to 25.2)	< 0.0001	0.28
Mean score for students^c^	34.7% (16.9%)	50.6% (23.6%)	16.0% (11.8 to 20.1%)		< 0.0001	0.24
**Teachers**^**d**^						
Teachers with a passing score (≥ 9/18)^b^	14 (40.0%)	31 (88.6%)	48.6% (29.4 to 67.8%)	12.1 (3.4 to 42.7)	< 0.0001	
Teachers with a mastery score (≥ 14/18)^b^	1 (2.9%)	20 (57.1%)	54.3% (37.0 to 71.6%)	45.3 (5.6 to 369.5)	< 0.0001	
Mean score for teachers^c^	43.3% (14.9%)	75.2% (17.5%)	31.9% (24.4 to 39.4%)		< 0.0001	

Data are % (SD), % (95% CI), or *n* (%)

*ICC* intraclass correlation coefficient

^a^The cluster design was accounted for using random intercepts at the level of school

^b^Logistic regression was used to estimate an adjusted odds ratio, which is re-expressed as an adjusted risk difference

^c^Linear regression was used to estimate an adjusted difference in means

^d^Teachers were treated as equivalent to the units of randomization (schools), so these models did not include random intercepts. The stratification variable was modeled as a fixed effect in all analyses. Wald-type confidence intervals and two-sided normal *p* values were computed in all analyses

In the intervention arm, 228/1238 (18.4%) of the students had a mastery score compared to 28/1181 (2.4%) in the control arm (adjusted odds ratio 11.7 [95% CI 5.4–25.2], *p* < 0.0001). The adjusted difference was 14.3% (95% CI 9.5–19.1%). The mean test score among students in the intervention arm was 50.6% (SD: 23.6%) compared to 34.7% (SD: 16.9%) in the control arm (adjusted mean difference 16.0% [95% CI 11.8–20.1%], *p* < 0.0001).

Among the teachers who completed the test after 1 year, 31/35 (88.6%) had a passing score in the intervention arm compared to 14/35 (40.0%) in the control arm (adjusted odds ratio 12.1 [3.4–42.7], *p* < 0.0001). The adjusted difference was 48.6% (95% CI 29.4–67.8%). In the intervention arm, 20/35 (57.1%) of the teachers had a mastery score compared to 1/35 (2.9%) in the control arm (adjusted odds ratio 45.3 [5.6–368.5], *p* < 0.0001). The adjusted difference was 54.3% (95% CI 37.0–71.6%). The mean test score among teachers in the intervention arm was 75.2% (SD: 17.5%) compared to 43.3% (SD: 14.9%) in the control arm (adjusted mean difference 31.9% [95% CI: 24.4–39.4%], *p* < 0.0001).

### Performance of students on each concept

**Fig. 2:**
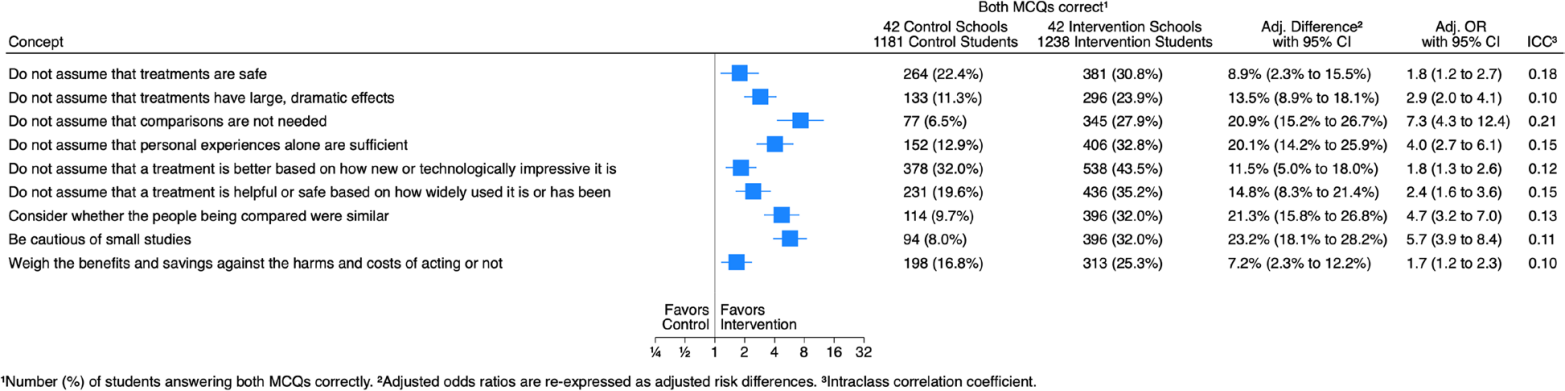
Results for each key concept covered in the 1-year follow-up trial. *p* < 0.0001 for all comparisons. ^1^Number (%) of students answering both MCQs correctly. ^2^Adjusted odds ratios are re-expressed as adjusted risk differences. ^3^Intraclass correlation coefficient

Students who answered correctly both questions for each of the nine concepts were better in the intervention arm compared to the control arm for all the concepts (Fig. 2). The adjusted difference was largest for the concept “Be cautious of small studies.” For that concept, 396/1238 (32.0%) answered both questions correctly in the intervention arm compared to 94/1181 (8.0%) in the control arm (adjusted difference 23.2% [95% CI 18.1–28.2%]). The adjusted difference was smallest for the concept “Weigh the benefits and savings against the harms and costs of acting or not.” For that concept, 313/1238 (25.3%) answered both questions correctly in the intervention arm compared to 198/1181 (16.8%) in the control arm (adjusted difference 7.2% [95% CI: 2.3–12.2%]).

### Subgroup analysis on school performance and English proficiency

The effect of the intervention was similar in the high and low performing schools. The odds ratio of the interaction between school performance and the intervention was 0.9 (95% CI 0.3–2.6, *p* = 0.89) (Table 3).

**Table 3:** Subgroup analyses on school performance and English proficiency

	**Control schools (*n* = 42)**	**Intervention schools (*n* = 42)**	**Adjusted difference**	**Adjusted odds ratio**	***p* value**	**ICC**
**Low and high performing schools**						
**Low performing schools**	1010 students 24 schools	1010 students 24 schools				
Students with a passing score (≥ 9/18)	48 (7.6%)	236 (34.9%)	26.7% (18.1 to 35.3%)	7.7 (4.0 to 14.9)	< 0.0001	0.20
**High performing schools**	921 students 18 schools	896 students 18 schools				
Students with a passing score (≥ 9/18)	182 (33.3%)	389 (69.2%)	38.6% (24.8 to 52.3%)	7.6 (3.4 to 16.8)	< 0.0001	0.28
**Interaction**						
Intervention × high performance				0.9 (0.3 to 2.6)	0.8950	
**Students with advanced, basic, and lacking English reading proficiency**						
**Advanced proficiency**	745 students 42 schools	755 students 42 schools				
Students with a passing score (≥ 9/18)	119 (32.2%)	317 (75.3%)	42.7% (32.2 to 53.1%)	10.2 (5.3 to 19.6)	< 0.0001	0.25
**Basic proficiency**	709 students 42 schools	679 students 42 schools				
Students with a passing score (≥ 9/18)	68 (20.4%)	195 (56.5%)	37.4% (27.5 to 47.2%)	9.4 (4.8 to 18.5)	< 0.0001	0.26
**Lacking proficiency**	852 students 42 schools	806 students 42 schools				
Students with a passing score (≥ 9/18)	43 (9.0%)	113 (23.9%)	15.8% (8.7 to 22.8%)	3.9 (2.1 to 7.3)	< 0.0001	0.21
**Interactions with reading proficiency**						
Intervention × basic proficiency			0.7 (0.4 to 1.2)	0.1965		
Intervention × lacking proficiency			0.3 (0.2 to 0.5)	< 0.0001		
Joint test of no interaction					0.0002	

Data are *n* (%) and % (95% CI)

*ICC* intraclass correlation coefficient

Logistic regression was used to estimate adjusted odds ratios, which are re-expressed as adjusted risk differences. The cluster design was accounted for using random intercepts at the level of school. Wald-type confidence intervals and two-sided normal *p* values were computed in all analyses. Low school performance was used as the reference and advanced English reading proficiency was used as the reference

The effect of the intervention was also similar for students with advanced and basic English reading proficiency (OR 0.7 [95% CI 0.4–1.2], *p* = 0.19) (Table 3). Students who lacked English reading proficiency were less likely to achieve a passing score compared to those with advanced reading proficiency (OR 0.3 [95% CI 0.2–0.5], *p* < 0.0001). However, the intervention was effective for students who lacked English proficiency (OR 3.9 [95% CI 2.1–7.3], *p* < 0.0001).

### Self-efficacy and intended behaviors of students participated in the trial after 1 year

There were only small differences in the proportion of students in the intervention and control arms who found it easy or very easy to know if a claim about treatment was based on a research study comparing treatments (adjusted difference 7.8% [95% CI 2.1 to 13.5%]), to find information about treatments that is based on research (adjusted difference 3.4% [95% CI − 2.5 to 9.2%]), to judge the trustworthiness of the results of a research study comparing treatments (adjusted difference 3.0% [95% CI − 2.6 to 8.6%]), or to judge the relevance of a research study comparing treatments (adjusted difference 5.5% [95% CI − 0.2 to 11.1%]) (Table S1).

There also were only small differences in the proportion of students in the intervention and control arms who were likely or very likely to find out the basis of the claim (adjusted difference 0.1% [95% CI − 5.6 to 5.9%]), to find out if a claim was based on a research study (adjusted difference 5.0% [95% CI − 0.6 to 10.7%]), or to participate in the research study if asked (adjusted difference 0.9% [95% CI − 3.7 to 5.6%]) (Table S2).

Most students in the intervention arm liked the lessons a little or very much (82.5%), found the lessons easy or very easy to understand (66.6%), and found what they learned helpful or very helpful (84.7%) (Table S3).

### Sensitivity analysis

There were only small differences between the unweighted analysis and the analysis done using inverse probability weighting for students for passing scores (adjusted difference 32.2% vs 31.8%), mastery scores (adjusted difference 14.3% vs 13.7%), and the mean difference (16.0% vs 15.7%) (Tables 2 and 4).

**Table 4:** Sensitivity analysis

	**Control schools**	**Intervention schools**	**Adjusted difference**	**Adjusted odds ratio**	***p* value**	**ICC**
	42 schools 1181 students 35 teachers	42 schools 1238 students 35 teachers				
**Students**						
**Primary outcome**^**a**^						
Students with a passing score (≥ 9/18)^b^	230 (19.5%)	625 (50.5%)	31.8% (24.2 to 39.4%)	7.8 (4.6 to 13.1)	< 0.0001	0.26
**Secondary outcomes**^**a**^						
Students with a mastery score (≥ 14/18)^b^	28 (2.4%)	228 (18.4%)	13.7% (8.8 to 18.5%)	11.9 (5.3 to 26.7)	< 0.0001	0.32
Mean score for students^c^ Lee bounds^d^	34.7% (16.9%)	50.6% (23.6%)	15.7% (11.6 to 19.9%) 14.2 to 17.5% (7.6% to 23.3%)		< 0.0001	0.25
**Teachers**^**e**^						
Teachers with a passing score (≥ 9/18)^b^	14 (40.0%)	31 (88.6%)	48.6% (29.4 to 67.8%)	12.1 (3.4 to 42.7)	1.0e − 04	
Teachers with a mastery score (≥ 14/18)^b^	1 (2.9%)	20 (57.1%)	54.3% (37.0 to 71.6%)	45.3 (5.6 to 369.5)	3.7e − 04	
Mean score for teachers^c^ Lee bounds^d^	43.3% (14.9%)	75.2% (17.5%)	31.9% (24.4 to 39.4%) Not estimable		< 0.0001	

Data are % (SD), % (95% CI), or *n* (%)

*ICC* intraclass correlation coefficient

^a^The cluster design was accounted for using random intercepts at the level of school

^b^Logistic regression was used to estimate an adjusted odds ratio, which is re-expressed as an adjusted risk difference

^c^Linear regression was used to estimate an adjusted difference in means

^d^Interval estimates provide sharp bounds on treatment effect under conditions in which missing outcome data maximally favor or disfavor the intervention; cluster-randomization is accounted for via design effect adjustments.

^e^Teachers were treated as equivalent to the units of randomization (schools), so these models did not include random intercepts. Inverse probability weighting was used to account for missing outcome data, and the stratification variable was modeled as a fixed effect in all analyses. Wald-type confidence intervals and two-sided normal *p* values were computed for all analyses

The Lee bounds were 14.2 to 17.5% for the adjusted difference in mean scores for students (95% CI 7.6–23.3%) (Table 4). It was not possible to calculate Lee bounds for the adjusted difference in mean scores for teachers because the number of teachers lost to follow-up was the same in the control and intervention arms.

## Discussion

After 1 year, the IHC secondary school intervention was effective in improving students’ critical thinking about health claims compared to the usual curriculum. The effect for the primary outcome (passing scores) was smaller after 1 year compared to the end of the term when the intervention was delivered (adjusted difference 32.2% vs 37.2%). This was due to decay in the proportion of students in the intervention schools with a passing score compared to the end of the intervention term. Retention was 86.8% (50.5% passing in intervention schools after 1 year compared to 58.2% at the end of the intervention term) [22]. The proportion of students with a passing score in control schools was about the same at the end of the intervention term and after 1 year (19.5% vs 19.4%).

There also was a reduction in the proportion of students in the intervention schools with a mastery score and the size of the effect for mastery scores after 1 year compared to the end of the intervention term (adjusted difference 14.3% vs 22.3%). Retention was 78.3% (18.4% mastery in intervention schools after 1 year compared to 23.5% at the end of the intervention term). The effect on the mean score also was smaller after 1 year (adjusted difference 16.0% vs 20.8%). Retention for students in intervention schools was 91.3% (50.6% after 1 year compared to 55.4% at the end of the intervention term).

For teachers, the effect on passing scores was slightly less after 1 year (adjusted difference 48.6%) compared to the end of the intervention term (adjusted difference 50.0%). For mastery scores, the adjusted difference between the intervention and control schools was 54.3% after 1 year compared to 71.4% at the end of the intervention term. The adjusted difference for mean scores was 31.9% after 1 year compared to 36.9% at the end of the intervention term.

Both at the end of the intervention term and after 1 year, the intervention had similar effects in schools categorized as low and high performing. Students who had low English reading proficiency benefitted less from the intervention than students with advanced reading proficiency. Responses to the questions about self-efficacy, intended behaviors, and students’ perceptions of the lessons also were slightly similar at the end of the intervention term and after 1 year.

The parallel 1-year follow-up studies in Kenya and Uganda had similar results (unpublished work). The proportion of students with a passing score in Kenya was 53.2% in intervention schools compared 32.2% in control schools after 1 year (adjusted difference 21.2%, 95% CI 14.1–28.3). In Uganda, the proportion of students with a passing score after 1 year was 53.4% compared to 33.1% (adjusted difference 21.9%, 95% CI 17.1–32.8%). Overall, across the three studies, 52.6% of students in intervention schools had a passing score after 1 year compared to 58.1% at the end of the intervention term (retention 90.5%). When adjusted for chance, retention was 88.3%. Taken together, these studies suggest that the findings are broadly applicable to schools that follow the national curriculum in East Africa. The extent to which the findings are applicable to schools that are hard to reach, special needs schools, schools that do not follow the national curriculum, and schools in other countries is uncertain.

A similar 1-year follow-up study of the effects of the IHC primary school intervention [9] found that the proportion of students with a passing score in intervention schools increased from 69.0% at the end of the intervention term to 80.1% after 1 year. However, the proportion of students in control schools with a passing score increased even more (from 26.8 to 51.5%), so the effect was smaller after 1 year (adjusted difference 39.5% vs 49.8%). No other randomized trials of school-based interventions to teach critical thinking about health have been reported results after 1 year [38]. More broadly, a review of long-term retention of basic science knowledge found that decay in what students learn in school is common [39, 40].

A limitation of this study is the loss to follow-up. Overall, 22.7% of students who participated in the trial did not complete the CTH test after 1 year (24.1% loss to follow-up in control schools and 21.2% in the intervention schools). Loss to follow-up was 16.6% for teachers in both arms of the trial. However, based on the pre-specified sensitivity analyses we conducted, the loss to follow-up is unlikely to have substantially biased the effect estimates. The main reasons for both teachers and students being lost to follow-up were absence from school on the day of the test was administered and change of school.

There were more teachers with a bachelor’s degree in the intervention schools compared to the control schools (61.9% vs 35.7%). This is unlikely to have biased the results for students, since critical thinking about health was not taught in control schools. We cannot rule out that it biased the results for teachers, but this seems unlikely since critical thinking is not included in the bachelor’s curriculum. It also is unlikely that it affected the applicability of the results. None of the teachers had taught critical thinking about health previously. All the teachers in intervention schools participated in the teacher training workshops, and all 42 teachers reported that the teacher training helped them to acquire the knowledge and skills needed to deliver the intervention [26].

Other limitations of this study are the same as those discussed in our report of the results at the end of the intervention term [20]. Responses to the questions about self-efficacy, intended behaviors, and students’ perceptions of the lessons may have been biased to some extent by social desirability, and that the Critical Thinking about Health Test was a treatment-inherent outcome measure.

## Conclusion

The 1-year follow-up results and the initial results show that it is possible to teach critical thinking about health in Rwandan secondary schools. Understanding and the ability to apply the key concepts covered in the lessons was retained for at least a year by half of the students. However, there is a need to improve retention and the proportion of students who benefit from the lessons. One way of doing this would be to introduce subsequent lessons to reinforce what was learned and introduce new concepts. Another would be to modify the delivery of these lessons by, for example, increasing the amount of time allocated to teaching the lessons, providing students with direct access to printed or digital materials, integrating the lessons into the usual curriculum, and examining students’ ability to apply the concepts in national evaluations.

## Supplementary Information

Supplementary Material 1.

## Data Availability

All de-identifiable individual-participant data and the data dictionary will be made available on Zenodo. The study protocol with the detailed analysis plan can be found online at https://doi.org/10.5281/zenodo.6562788.

## Abbreviations

CTH: Critical Thinking about Health Test
IHC: Informed Health Choices
NESA: National Examination and School Inspection Authority
PACTR: Pan African Clinical Trial Registry
RNEC: Rwanda National Ethics Committee
