# Supplementary material for: What is the effect of the Informed Health Choices secondary school intervention on the ability of students in Rwanda to think critically about health choices after one-year follow-up? A cluster-randomized trial
Source: Trials. 2025 May 15;26:160. doi: 10.1186/s13063-025-08779-w (PMC12082997; doi:10.1186/s13063-025-08779-w)
Supplement: Supplementary file 1 — Supplementary Material 1. [file 13063_2025_8779_MOESM1_ESM.docx]

**Supplementary files**

Supplementary file 1: GREET checklist. 2

Supplementary file 2: The Critical Thinking about Health Test 8

Table S1: Self-efficacy of participants in control and intervention arm. 29

Table S2: Intended behaviors of participants in control and intervention arm. 30

Table S3: Intervention students’ views on the lessons covered in the trial. 31

# Supplementary file 1: GREET checklist.

Guideline for reporting evidence-based practice educational interventions and teaching (GREET) checklist^1^.

BRIEF NAME

1. **Intervention:** Informed Health Choices (IHC) secondary school intervention

The intervention was compared to routine practice (teaching according to the national lower-secondary school curriculum without intervening).

WHY this educational process

2. **Theory:** The IHC secondary school resources are based on the *IHC Key Concepts* framework. The framework includes concepts (principles) that people should understand and apply when deciding whether to believe a claim about the effects of health actions (things that people do to care for their health or the health of others) and what to do.^2,3^ The framework is based on evidence of the importance of the included concepts,^4,5^ logic, feedback, other relevant frameworks, ^6^ and adaptation of the IHC Key Concepts to other types of interventions such as educational, environmental, and policing interventions.^7^

The resources were developed by the investigators between 2020 and 2022 using human-centred design methods.^8^ This included cycles of idea generation and prototyping, piloting with observation, user-testing with teachers and students, and feedback from teachers, students, and curriculum developers in Kenya, Rwanda, and Uganda, and an international advisory group. The aim of the design process was to ensure that teachers and students find the resources to be engaging, useful, and easy to use.

The teaching strategies used in the resources were based in part on an overview of systematic reviews of teaching strategies, ^9^ and draw on several educational theories. These include social constructivist theory (which postulates that learning can be maximized through well-designed, intentional social interaction with other learners), ^10^ the theory of active student response (which postulates that learning is enhanced by high levels of active student response), ^11^ and the elaborative retrieval hypothesis (which postulates that the search for correct answers on practice tests or quizzes results in multiple retrieval routes which aid later recall). ^12^

3. **Learning objectives:** The primary learning goal is for students to have a basic ability to think critically about health actions and understand why this is important. They should be able to recognise claims about the effects of health actions and assess some of those claims. They should understand why it is important for them that researchers study the effects of health actions and recognise two key features of reliable comparisons of health actions. They should recognise that health actions can have both advantages and disadvantages and the importance of weighing the benefits and savings against the harms and costs when deciding what to do.

4. **Evidence-based practice content:** The resources focus on nine IHC Key Concepts that were prioritised by curriculum developers, teachers, and researchers in Kenya, Rwanda, and Uganda. ^13^

WHAT

5. **Materials:** The IHC secondary school resources ([*Be smart about your health*](https://besmarthealth.org/)) are open access digital resources for lower-secondary school teachers. The 10 lessons are provided as lesson plans in two formats: for teachers who are using either a blackboard and or a projector in the classroom. The aim is for students to learn to think critically about health claims and choices. The resources were made available to schools in the intervention group. Teachers in those schools downloaded the resources to a computer or smartphone and delivered the lessons. Schools in both the control and intervention group continued teaching the national curriculum, which did not include teaching critical thinking about health. No additional materials were provided to the control schools.

Each Lesson includes an introduction, an activity, and a wrap-up. The introduction includes the key messages from the previous lesson, a question about the previous lesson, and what this lesson is about. The activity is designed to help students achieve the learning goals. The wrap-up includes a question about what was learned, the key messages for the lesson, a homework assignment, if there is one, and what the next lesson is about. Lessons 5 and 10 include quizzes and discussions of application of what students learned in their daily lives.

For each of the 10 lessons there is an overview and background for teachers. The overview includes learning goals, key terms introduced in the lesson, and the main teaching strategies used in the lesson. The background includes a description of what the lesson is about and if relevant, common misunderstandings and closely related content that is not covered in the lesson.

In addition, there is a teachers’ guide, materials for teacher training workshops, information about how to use the resources (help), optional printouts (PDFs) for teachers and students, and a glossary. Teachers were provided with binders with printouts at the training workshops.

6. **Educational strategies:** Key strategies used across lessons included guided note taking, small group discussion, use of response cards, ^11^ homework, use of a standard lesson structure, setting objectives and providing feedback, and multimedia design. Other strategies used in some of the lessons include concept cartoons, inquiry-based instruction, and role play.

7. **Incentives:** The incentive for teachers and students was the value they perceived in learning to think critically about health actions. Teachers at schools without Internet access were reimbursed for the cost of downloading the resources and any other costs related to participation in the trial. They were not paid for participating in the trial and there were no other financial incentives for the schools, head teachers, teachers, or students. The evaluation administered at the end of the school term did not count towards the students’ school marks or assessment of the teachers or schools.

WHO PROVIDED

8. **Instructors**: The head teacher at each participating school selected a teacher of a relevant subject (e.g., biology) for year-1 or year-2 of lower-secondary school. The teachers were invited to a 2-3-day workshop to introduce them to the resources and the learning content. The training was facilitated by other teachers who had participated in one of the teacher networks that helped to develop the resources or who piloted use of the resources. The facilitators were provided with presentations and other materials for the workshops, and they reviewed the material and plans for the workshops with the research teams prior to the workshops.

HOW

9. **Delivery:** The 10 lessons were delivered by the teachers during regular classroom time or, if necessary, outside of regular classroom time. They could use a computer, smartphone, or printouts when delivering the lessons. Depending on what equipment was available to the teachers, they delivered the lessons to students using only a blackboard or using a projector and slide presentations that are included in the digital resources. The number of students in a class varied.

WHERE

10. **Environment:** Representative samples of schools were recruited, including rural and urban schools. The conditions in the schools varied. Details of the context can be found in report of the context analysis undertaken prior to developing the resources. ^14^

WHEN and HOW MUCH

11. **Schedule:** The 10 lessons were taught in a single school term. Each school decided how to fit the lessons into the schedule for that term.

12. **Amount of time**: Each lesson was designed to be delivered in a single period (40 minutes). The students were encouraged to collect and assess claims about the effects of health actions outside of class and to discuss claims with their families and friends. The teachers needed up to 30 minutes to prepare for each lesson.

PLANNED CHANGES

13. **Adaptation**: No specific adaptation was required, but teachers were able to adapt the lessons, for example by using different or additional examples or editing the presentations.

UNPLANNED CHANGES

14. **Modifications**: As part of the process evaluations, teachers were asked to complete an evaluation form after each lesson, including information about changes they made to the lesson plan, and some teachers were observed for one to two lessons. No feedback was given to the teachers during the trial.

HOW WELL

15. **Attendance:** The teachers were asked to record attendance for each lesson. Students were encouraged to attend all lessons by telling them when the next lesson would be and its learning goals. The lessons were designed to appeal to students and to make clear the relevance and importance of the learning goals.

16. **Fidelity**: We will explore the extent to which the lessons were delivered as planned in the process evaluation, based on the evaluation forms completed by teachers after each lesson, observations of their teaching a lesson, and interviews with teachers and students.

17. **Delivery schedule**: The teachers were asked to record when each lesson was taught, the duration of each lesson, and whether all the lesson were completed as planned.

**References**

1. Phillips AC, Lewis LK, McEvoy MP, et al. Development and validation of the guideline for reporting evidence-based practice educational interventions and teaching (GREET). *BMC Med Educ* 2016; **16**: 237.

2. Oxman AD, Chalmers I, Austvoll-Dahlgren A, Informed Health Choices Group. Key Concepts for assessing claims about treatment effects and making well-informed treatment choices. *F1000Res* 2019; **7**: 1784.

3. Oxman AD, Chalmers I, Dahlgren A, Informed Health Choices Group. Key Concepts for assessing claims about treatment effects and making well-informed treatment choices. Version 2019. *IHC Working Paper* 2019.

4. Oxman AD, Chalmers I, Dahlgren A, Informed Health Choices Group. Key Concepts for Informed Health Choices: a framework for enabling people to think critically about health claims (Version 2022). *IHC Working Paper* 2022.

5. Oxman AD, Chalmers I, Dahlgren A. Key Concepts for Informed Health Choices: Where’s the evidence? *F1000Res* 2022; **11**: 890.

6. Oxman AD, Martinez Garcia L. Comparison of the Informed Health Choices Key Concepts to other frameworks that are relevant to learning how to think critically about treatment claims, comparisons, and choices: protocol for a mapping review. *IHC Working Paper* 2018.

7. Aronson JK, Barends E, Boruch R, et al. Key concepts for making informed choices. *Nature* 2019; **572**(7769): 303-6.

8. Rosenbaum SE, Moberg J, Chesire F, et al. Teaching critical thinking about health information and choices in secondary schools: human-centred design of digital resources. *F1000Res* 2023.

9. Oxman AD, Nsangi A, Martinez Garcia L, et al. The effects of teaching strategies on learning to think critically in primary and secondary schools: an overview of systematic reviews. *Review of Education* Submitted 20 January 2023.

10. Igel C. Cooperative learning. Denver, CO: McREL International, 2010.

11. Randolph JJ. Meta-analysis of the research on response cards: effects on test achievement, quiz achievement, participation, and off-task behavior. *J Posit Behav Interv* 2007; **9**(2): 113-28.

12. Pan SC, Rickard TC. Transfer of test-enhanced learning: meta-analytic review and synthesis. *Psychol Bull* 2018; **144**(7): 710-56.

13. Agaba JJ, Chesire F, Mugisha M, et al. Prioritisation of Informed Health Choices (IHC) Key Concepts to be included in lower-secondary school resources: a consensus study. *PLoS One* 2023; **18**: e0267422.

14. Mugisha M, Uwitonze AM, Chesire F, et al. Teaching critical thinking about health using digital technology in lower secondary schools in Rwanda: A qualitative context analysis. *PLoS One* 2021; **16**(3): e0248773.

# Supplementary file 2: The Critical Thinking about Health Test

**Instructions**

*Before you start, please note that some words in this questionnaire may not be familiar to you. Please read through the following explanations:*

A **TREATMENT** is anything done to care for yourself, so you stay well or, if you are sick or injured, so you get better and not worse. For example, skin cream.

A **TREATMENT CLAIM** is something someone says about whether a treatment causes something to happen or to change. A claim can be true or can be false. For example, if a friend says “Using skin cream will help your skin rash”.

A **RESEARCH STUDY** is a way to answer a question by carefully collecting information. For example, a study might be done to answer the question: Does skin cream help people with skin rash?

**RESULTS of a study** are what the study found. For example, whether people who use skin cream had less skin rash.

When something happens by **CHANCE**, it is not possible to tell in advance what will happen. For example, if you flip a coin, you cannot tell in advance if it will land on one side or the other side.

First, read the text above the questions and then answer each question on **the SCORE sheet,** using one of the provided answers.

For each question, choose what you think is the best answer and

**fill in the circle** for that answer in the score sheet, like this.


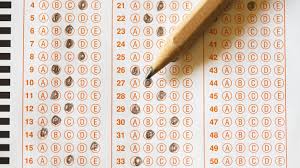


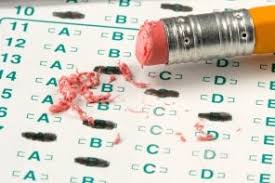


**If you want to change your answer,** carefully erase the first circle that you filled in.


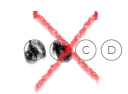
Do not fill in more than one circle for each question.

The examples below show you the one correct way and some wrong ways to mark your answers.

**Be sure to fill in the circles the correct way.**


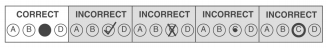


**Part 1.**

**Questions about you**

**1. District code**

**2. Your school code**

**2. Your school code**

**2. School code**

**3 Student code**

**4 Your age**

**5. Your gender** ☐ M

☐ F

| A doctor did a research study to find out if drinking tea keeps people from getting sick. He flipped a coin to decide who should get the tea and who should not. People who got tea went to the doctor’s office every day to drink their tea. At the end of the study, people who got the tea were less likely to be sick than those who got no tea.  *Based on the text above, please answer the following questions:* |
| --- |
| 6.  *Question*: Who went to the doctor’s office every day? |
| *Options:*   1. People who did not get tea 2. People who got tea 3. Everyone 4. People who got sick |
|  |
| 7.  *Question*: How did the doctor decide who should get tea? |
| *Options:*   1. By flipping a coin 2. By asking people if they would like tea 3. The doctor gave tea to those who were more likely to be sick 4. The doctor asked people who came to his office |
|  |

**Part 2. Reading ability questions**

| A doctor did a research study to find out if drinking tea keeps people from getting sick. He flipped a coin to decide who should get the tea and who should not. People who got tea went to the doctor’s office every day to drink their tea. At the end of the study, people who got the tea were less likely to be sick than those who got no tea.  *Based on the text above, please answer the following questions:* |
| --- |
| 8. *Question*: **What was the treatment?** |
| *Options:*   1. Tea 2. Sleep 3. The study 4. The doctor |
|  |
| **9.** *Question*: **What was the result of the study?**  *Options:*   1. Drinking tea can help people from getting sick 2. Doctors should toss coins when doing studies 3. People should go to the doctor if they are sick 4. Not drinking tea can help people from getting sick |
|  |

**Part 3.**

**Questions about claims**

| 10. *Question*: | |
| --- | --- |
| Anne has pain in her ear, and she asks her brother Hassan what to do about it. He says that once, when he had a pain like that, he cleaned his ear with hot water. The next day, his ear pain was gone. Based on his experience, he says rinsing with hot water is helpful for ear pain. | |
| *Question:* Do you agree with Hassan? | |
| *Options:*   1. Yes. Because this is Hassan’s experience, it is likely to be true 2. No, Hassan’s experience is not enough to be sure 3. Yes, Hassan rinsed his ear with hot water and the next day his ear pain was gone | |
| 11. *Question*: | |
| Sarah says that medicines from well-known companies, costing more money, are not necessarily the best. Medicines from less known companies, costing less money, may be just as good or even better. | |
| *Question:* Is Sarah right? | |
| *Options:*   1. No, medicines costing less money are more likely to be harmful than expensive medicines 2. Yes, just because the medicine is expensive does not mean that it will work better than other medicines 3. No, expensive medicines made by well-known companies are better than less expensive medicines made by lesser-known companies | |
| 12. *Question*: | |
| Edith has stomach pain. Edith’s mother says that fruit juice is a good treatment for stomach pain. She learnt about this treatment from Edith’s grandmother. Over many years, other families she knows have also used fruit juice to treat stomach pain. | |
| *Question:* Based on this, how sure can we be that fruit juice is a good treatment for stomach pain? | |
| *Options:*   1. Not very sure. Even though people have used fruit juice over many years, that does not mean that it helps stomach pain 2. Very sure. If it has worked for Edith’s mother and other people who have tried it, it will probably work for her too 3. Not very sure. Edith should ask more families if they use fruit juice to treat stomach pain | |
| 13. *Question*: | |
| John has a skin rash on his leg. A shop sells several skin creams to treat skin rashes. John chooses a skin cream from a well-known company, even though it is more expensive than the other creams. John thinks this skin cream is more likely to heal his rash than the other skin creams because it is more expensive. | |
| *Question:* Is John right? | |
| *Options:*   1. No, just because the skin cream is expensive does not mean that it will work better than other creams 2. It is not possible to say. However, expensive skin creams are likely to be better because the companies spend more time making them 3. No, the skin cream is probably not as good as the other skin creams. People just like well-known companies more | |
| 14. *Question*: |  |
| Sarah has a sickness. There is a medicine for it, but she is not sure if she should try it. A research study comparing the medicine with no medicine found that the medicine was helpful but also that it could be harmful. Three of Sarah’s friends are telling her what to do. |  |
| *Question:* Which of the following things said by her friends is more correct? |  |
| *Options:*   1. She should only take the medicine if many people have tried the medicine before 2. She should only take the medicine if she thinks it will help her more than it will harm her 3. If Sarah has enough money to buy the medicine, it could not hurt to try it |  |
| 15. *Question*: |  |
| Imagine you and your friends have formed a team to take part in a local running competition. People on the other teams all had bananas for breakfast. You and your friends did not have bananas for breakfast and lost the race. Some people say that this was because your team had bread for breakfast and that made them run slower. |  |
| *Question:* If you did a research study comparing people who eat bananas for breakfast with people who don’t eat bananas for breakfast, how would you decide who should have bananas for breakfast? |  |
| *Options:*   1. By chance (like flipping a coin) to make sure the two groups are as similar as possible 2. By having the teams decide, to make it as fair as possible 3. By having the teachers decide, because they know who would benefit best from eating bananas |  |

| 16. *Question*: |
| --- |
| Regina has a sickness that makes it difficult for her to breathe. She hears on the radio about a medicine that has helped many people with breathing problems. |
| *Question:* How sure can Regina be that the medicine does not have any harms? |
| *Options:*   1. It is not possible to say, it depends on how much hope Regina has in the medicine 2. Very sure, since the medicine has helped many people, it is unlikely that it also harms people 3. Not very sure, because all medicines may harm people as well as help them |
| 17. *Question*: |
| Outside the city where Paul lives there are many farms. The farmers often get coughs. For many years, the farmers have used strong tea to treat their coughs. They say that the tea is good for them and that it protects them from becoming more sick.  Paul says that the farmers may not be right, and that the strong tea may not help coughs. |
| *Question:* Do you agree with Paul? |
| *Options:*   1. Yes, Paul should try drinking strong tea himself to know for sure. The strong tea may work differently on him 2. Yes, we can only know for sure if the strong tea works if it has been compared with other treatments in studies 3. No, the farmers would not have used strong tea for all those years if it did not work |

| 18. *Question*: |
| --- |
| Jane often has headaches. Her doctor tells her that there is a medicine that may help her, but it may harm her. The medicine is also very expensive. |
| *Question:* What does Jane need to think about before using the medicine? |
| *Options:*   1. If the medicine will help her more than it will hurt her, and if she thinks it is worth paying so much money for it 2. If anybody she knows has tried the medicine so that she can ask them what they thought about it 3. If she should ask another doctor, since the doctor must be wrong. A medicine which is helpful cannot be harmful |
| 19. *Question*: |
| Mercy wanted to know if eating bananas makes you run faster. To find out, she invited her six best friends to take part in a research study. Three friends each got bananas, and three friends did not get bananas. At the end of the study, the friends who did not get bananas ran a lot faster. |
| *Question:* How sure can Mercy be about her study’s results? |
| *Options:*   1. More sure, because Mercy found a difference between the groups in how fast they ran. This means that the study included enough people. 2. Less sure, because the difference between the two groups could have occurred by chance 3. More sure, if she repeats the study with six more friends |

| 20. *Question*: |
| --- |
| Doctors studied people with stomach pain before and after they took a new medicine. After taking the new medicine, many people felt less pain. |
| *Question:* Can we be sure that the new medicine is good for treating stomach pain? |
| *Options:*   1. No, taking the new medicine should have been compared either with not taking the medicine, or with taking an older medicine 2. Yes, people were asked how much pain they felt before and after they took the new medicine 3. Yes, the study was done by doctors |
| 21. *Question*: |
| A new and an old mosquito spray (insecticide) were compared in a research study. In the study, two houses were sprayed with the new spray, and two houses were sprayed with the old spray. Based on this study, the new spray was better for protecting against mosquito bites than the old spray. Neither of the sprays was found to be harmful to people. |
| *Question:* How sure can you be about what the study found? |
| *Options:*   1. Less sure, because only four houses were studied and the differences between sprays may have happened by chance 2. More sure, because the new spray was better for protecting against mosquito bites and it was not harmful 3. More sure, because the new spray was found to be better, and the differences between sprays is unlikely to have happened by chance |

| 22. *Question*: |
| --- |
| On the radio, there is someone selling a treatment - a new juice. The seller says that if you drink one glass of it every day, you will not get sick. |
| *Question:* How sure can you be that the new juice will keep you from getting sick? |
| *Options:*   1. It is not possible to say. I would have to try the new juice myself to be sure 2. Very sure, otherwise this news would not be on the radio 3. Not very sure. Very few treatments work so well |
| 23. *Question*: |
| Dr. Javier has done a research study giving a new medicine to people who were vomiting. Some of the people stopped vomiting after they got the new medicine. Dr. Javier says that this means that the medicine works. |
| *Question:* Is Dr. Javier right? |
| *Options:*   1. No. The people who used the medicine were not compared with similar people who did not use the medicine 2. Yes, some of the people stopped vomiting 3. No, since not all the people stopped vomiting |

| 24. *Question*: |
| --- |
| George has stomach pain. The last time George had a stomach pain was two months ago. That time, he drank some hot milk and after an hour, his stomach pain was gone. Therefore, George says hot milk cures stomach pain. |
| *Question:* Is George right? |
| *Options:*   1. It is not possible to say. His stomach pain might have gone away without the hot milk 2. It is not possible to say, but it is likely to be true based on the fact that George had this experience 3. Yes, George’s experience is enough to show that hot milk makes stomach pain go away |
| 25. *Question*: |
| Esther recommends a new treatment – a medicine - for pain. She says that everyone who has tried it felt better. |
| *Question:* How sure can you be that what Esther says about the new medicine is true? |
| *Options:*   1. Not very sure. Very large benefits, where everyone or nearly everyone gets better because of a treatment are rare 2. It is not possible to say. To be sure I would have to try the medicine for myself 3. Very sure. The medicine must be very good since everyone who has tried it got better |

| 26. *Question*: | |
| --- | --- |
| A doctor wanted to know which of two treatments was best for headaches. In a study to find out, he asked people to choose which treatment they would like to get. He compared the people who took each of the two treatments. | |
| *Question:* How sure can we be about the results of this comparison of the two treatments? | |
| *Options:*   1. More sure, because the doctor asked people to choose which treatment they wanted 2. Less sure, because the doctor should have decided who got which treatment 3. Less sure, because the doctor should have given people one of the two treatments by chance (like flipping a coin) | |
| 27. *Question*: |  |
| Mary wanted to find out which plants were best for treating people with headaches, so she did a research study to compare green plants with yellow plants. The people who used the green plants had fewer headaches compared to the people who used the yellow plants. |  |
| *Question:* How sure can we be that green plants are better than yellow plants? |  |
| *Options:*   1. It is not possible to say. Mary did not study possible harms of the plants 2. Very sure, since people who used the green plants had fewer headaches 3. Not very sure, it depends on how much people believe the green plants will work |  |

**Part 4. Questions about your views**

Below are some questions about what you think. **There are not right or wrong answers to these questions.**

Below are some actions. Please read each one carefully and give the answer that comes closest to how difficult or easy you find each of the actions to be. There are not right or wrong answers to these questions.

28. *Question:* How difficult or easy do you find knowing if a claim about a treatment is based on a research study comparing treatments?

*Options:*

1. Very difficult
2. Difficult
3. Easy
4. Very easy
5. I don’t know

29. *Question:* How difficult or easy do you think it is to find information about treatments that is based on research studies comparing treatments?

*Options:*

1. Very difficult
2. Difficult
3. Easy
4. Very easy
5. I don’t know

Below are some actions. Please read each one carefully and give the answer that comes closest to how difficult or easy you find each of the actions to be. There are not right or wrong answers to these questions.

30. *Question:* How difficult or easy do you find judging the trustworthiness of the results of a research study comparing treatments?

*Options:*

1. Very difficult
2. Difficult
3. Easy
4. Very easy
5. I don’t know

31. *Question:* How difficult or easy do you find knowing if the results of a research study comparing treatments are relevant to you?

*Options:*

1. Very difficult
2. Difficult
3. Easy
4. Very easy
5. I don’t know

Think about a sickness that you might get. Imagine someone claiming (saying) that a treatment might help you get better.

32. *Question:* How likely are you to find out what the claim was based on (for example by asking the person making the claim)?

*Options:*

1. Very unlikely
2. Unlikely
3. Likely
4. Very likely
5. I don’t know

33. *Question:* How likely are you to find out if the claim was based on a research study comparing the treatment to no treatment?

*Options:*

1. Very unlikely
2. Unlikely
3. Likely
4. Very likely
5. I don’t know

34. *Question:* How likely are you to say “yes” if you are asked to participate in a research study comparing two treatments for your sickness?

*Options:*

1. Very unlikely
2. Unlikely
3. Likely
4. Very likely
5. I don’t know

**Part 5.**

**Questions about your experience with the Be Smart about Your Health lessons**

Below are some questions about what you think. **There are not right or wrong answers to these questions.**

| 35. *Question:* How much did you like or dislike the lessons? |
| --- |
| *Options:*   1. I liked the lessons very much 2. I liked the lessons a little 3. I disliked the lessons a little 4. I disliked the lessons very much |
| **36.** *Question:* **How easy or difficult were these lessons to understand?** |
| *Options:*   1. Very difficult to understand 2. Difficult to understand 3. Easy to understand 4. Very easy to understand |
| **37.** *Question:* **How helpful or unhelpful has what you have learned been to you?** |
| *Options:*   1. Very helpful to me 2. Helpful to me 3. Unhelpful to me 4. Very unhelpful to me |

| **38.** *Question:* **Compared to what you learned in other subjects, how helpful has what you learned in these lessons been to you?** |
| --- |
| *Options:*   1. Less helpful 2. About the same 3. More helpful |
| **39.** *Question:* **How much have you used what you learned from the lessons?** |
| *Options:*   1. Not at all 2. A little 3. A lot |

| **40.** *Question:* **How much did you question, or challenge things said by YOUR TEACHERS, based on what you learned in these lessons?** |
| --- |
| *Options:*   1. Not at all 2. A little 3. A lot |
| **41.** *Question:* **If you questioned or challenged YOUR TEACHERS, how did this make you feel?** |
| *Options:*   1. I did not challenge my teachers 2. Very bad 3. Bad 4. Neither bad nor good 5. Good 6. Very good |

| **42.** *Question:* **How much did you question, or challenge things said by YOUR PARENTS OR OTHER ADULTS AT HOME, based on what you learned in these lessons?** |
| --- |
| *Options:*   1. Not at all 2. A little 3. A lot |
| **43.** *Question:* **If you questioned or challenged YOUR PARENTS OR OTHER ADULTS AT HOME, how did this make you feel?** |
| *Options:*   1. I did not challenge my parents or other adults at home 2. Very bad 3. Bad 4. Neither bad nor good 5. Good 6. Very good |

| **44.** *Question:* **How much did you question, or challenge things said by OTHER STUDENTS OR FRIENDS,** **based on what you learned in the lessons?** |
| --- |
| *Options:*   1. Not at all 2. A little 3. A lot |
| **45.** *Question:* **If you questioned or challenged OTHER STUDENTS OR FRIENDS, how did this make you feel?** |
| *Options:*   1. I did not challenge other students or friends 2. Very bad 3. Bad 4. Neither bad nor good 5. Good 6. Very good |

| **46.** *Question:* **How stressful were the lessons for you?** |
| --- |
| *Options:*   1. Not at all stressful 2. A little stressful 3. Stressful 4. Very stressful |
| **47.** *Question:* **Did the lessons have any bad effects or disadvantages for you apart from stress or feeling bad about challenging what others said?** |
| *Options:*   1. Yes 2. No |

# Table S1: Self-efficacy of participants in control and intervention arm.

|  | **How difficult or easy would you find each of these actions to be?** | | | | | | | |
| --- | --- | --- | --- | --- | --- | --- | --- | --- |
|  | How difficult or easy do you find knowing if a claim about a treatment is based on a research study comparing treatments? | | How difficult or easy do you think it is to find information about treatments that is based on research studies comparing treatments? | | How difficult or easy do you find judging the trustworthiness of the results of a research study comparing treatments? | | How difficult or easy do you find knowing if the results of a research study comparing treatments are relevant | |
|  | **Control schools** | **Intervention schools** | **Control schools** | **Intervention schools** | **Control schools** | **Intervention schools** | **Control schools** | **Intervention schools** |
|  | **(1181 students)** | **(1238**  **students)** | **(1181 students)** | **(1238**  **students)** | **(1181 students)** | **(1238**  **students)** | **(1181 students)** | **(1238**  **students)** |
| **Missing** |  |  |  |  |  |  |  |  |
| **I don't know** | 150 (12.7%) | 156  (12.6%) | 105  (8.9%) | 122  (9.9%) | 126 (10.7%) | 148  (12.0%) | 130 (11.0%) | 128  (10.3%) |
| **Very difficult** | 253 (21.4%) | 228  (18.4%) | 245 (20.7%) | 225  (18.2%) | 263 (22.3%) | 225  (18.2%) | 192 (16.3%) | 161  (13.0%) |
| **Difficult** | 383 (32.4%) | 325  (26.3%) | 334 (28.3%) | 320  (25.8%) | 354 (30.0%) | 362  (29.2%) | 278 (23.5%) | 270  (21.8%) |
| **Easy** | 240 (20.3%) | 344  (27.8%) | 326 (27.6%) | 331  (26.7%) | 272 (23.0%) | 332  (26.8%) | 366 (31.0%) | 408  (33.0%) |
| **Very easy** | 155 (13.1%) | 185  (14.9%) | 171 (14.5%) | 240  (19.4%) | 166 (14.1%) | 171  (13.8%) | 215 (18.2%) | 271  (21.9%) |
| **Easy or very easy** | 395 (33.4%) | 529  (42.7%) | 497 (42.1%) | 571  (46.1%) | 438 (37.1%) | 503  (40.6%) | 581 (49.2%) | 679  (54.8%) |
| **Adjusted odds ratio¹** | 1.4  (95% CI 1.1 to 1.8) | | 1.2  (95% CI 0.9 to 1.5) | | 1.1  (95% CI 0.9 to 1.5) | | 1.3  (95% CI 1.0 to 1.6) | |
| **Adjusted difference²** | 7.8%  (95% CI 2.1% to 13.5%) | | 3.4%  (95% CI -2.5% to 9.2%) | | 3.0%  (95% CI -2.6% to 8.6%) | | 5.5%  (95% CI -0.2% to 11.1%) | |

¹Mixed-effects logistic regression was used to estimate adjusted odds ratios. The stratification variables were modelled as fixed effects, and the cluster design was accounted for using random intercepts at the level of school. ²Odds ratios are re-expressed as adjusted risk differences.

# Table S2: Intended behaviors of participants in control and intervention arm.

|  | Think about an illness that you might get. Imagine someone claiming (saying) that a particular treatment might help you get better. | | | | | |
| --- | --- | --- | --- | --- | --- | --- |
|  | How likely are you to find out what the claim was based on (for example by asking the person making the claim)? | | How likely are you to find out if the claim was based on a research study comparing the treatment to no treatment? | | How likely are you to say “yes” if you are asked to participate in a research study comparing two treatments for your sickness? | |
|  | **Control schools** | **Intervention schools** | **Control schools** | **Intervention schools** | **Control schools** | **Intervention schools** |
|  | **(1181 students)** | **(1238**  **students)** | **(1181**  **students)** | **(1238**  **students)** | **(1181**  **students)** | **(1238**  **students)** |
| **Missing** | 0 | 0 | 0 | 0 | 0 | 0 |
| **I don't know** | 97 (8.2%) | 83 (6.7%) | 124 (10.5%) | 98 (7.9%) | 112 (9.5%) | 96 (7.8%) |
| **Very unlikely** | 116 (9.8%) | 162 (13.1%) | 206 (17.4%) | 212 (17.1%) | 179 (15.2%) | 189 (15.3%) |
| **Unlikely** | 285 (24.1%) | 273 (22.1%) | 300 (25.4%) | 288 (23.3%) | 219 (18.5%) | 240 (19.4%) |
| **Likely** | 413 (35.0%) | 423 (34.2%) | 322 (27.3%) | 363 (29.3%) | 312 (26.4%) | 360 (29.1%) |
| **Very likely** | 270 (22.9%) | 297 (24.0%) | 229 (19.4%) | 277 (22.4%) | 359 (30.4%) | 353 (28.5%) |
| **Likely or very likely** | 683 (57.8%) | 720 (58.2%) | 551 (46.7%) | 640 (51.7%) | 671 (56.8%) | 713 (57.6%) |
| **Adjusted odds ratio¹** | 1.0 (95% CI 0.8 to 1.3) | | 1.2 (95% CI 1.0 to 1.6) | | 1.0 (95% CI 0.9 to 1.3) | |
| **Adjusted difference²** | 0.1% (95% CI -5.6% to 5.9%) | | 5.0% (95% CI -0.6% to 10.7%) | | 0.9% (95% CI -3.7% to 5.6%) | |

¹Mixed-effects logistic regression was used to estimate adjusted odds ratios. The stratification variables were modelled as fixed effects, and the cluster design was accounted for using random intercepts at the level of school. ²Odds ratios are re-expressed as adjusted risk differences.

# Table S3: Intervention students’ views on the lessons covered in the trial.

| **How much did you like or dislike the lessons?** | | **How easy or difficult were these lessons to understand?** | | **How helpful or unhelpful has what you have learned been to you?** | |  |  |
| --- | --- | --- | --- | --- | --- | --- | --- |
| **Intervention schools (1238 students)** | | | | | | | |
| **Liked very much** | 841 (67.9%) | **Very easy** | 171 (13.8%) | **Very helpful** | 721 (58.2%) |  |  |
| **Liked a little** | 180 (14.5%) | **Easy** | 224 (18.1%) | **Helpful** | 328 (26.5%) |  |  |
| **Disliked a little** | 96 (7.8%) | **Difficult** | 520 (42.0%) | **Unhelpful** | 105 (8.5%) |  |  |
| **Disliked a lot** | 101 (8.2%) | **Very difficult** | 305 (24.6%) | **Very helpful** | 65 (5.3%) |  |  |
| **Liked a little or very much** | 102 (82.5%) | **Easy or very easy** | 825 (66.6%) | **Helpful or very helpful** | 1049 (84.7%) |  |  |

Data are for participants who took the test after one year in the intervention arm.
